# Associations Between Mental Health, Interoception, Psychological Flexibility, and Self-as-Context, as Predictors for Alexithymia: A Deep Artificial Neural Network Approach

**DOI:** 10.3389/fpsyg.2021.637802

**Published:** 2021-03-24

**Authors:** Darren J. Edwards, Rob Lowe

**Affiliations:** ^1^Department of Public Health, Policy, and Social Sciences, Swansea University, Swansea, United Kingdom; ^2^Department of Psychology, Swansea University, Swansea, United Kingdom

**Keywords:** psychological flexibility, process based therapy, interoception, alexithymia, self-as-context

## Abstract

**Background:** Alexithymia is a personality trait which is characterized by an inability to identify and describe conscious emotions of oneself and others.

**Aim:** The present study aimed to determine whether various measures of mental health, interoception, psychological flexibility, and self-as-context, predicted through linear associations alexithymia as an outcome. This also included relevant mediators and non-linear predictors identified for particular sub-groups of participants through cluster analyses of an Artificial Neural Network (ANN) output.

**Methodology:** Two hundred and thirty participants completed an online survey which included the following questionnaires: Toronto alexithymia scale; Acceptance and Action Questionnaire 2 (AQQII); Positive and Negative Affect Scale (PANAS-SF), Depression, Anxiety, and Stress Scale 21 (DAS21); Multidimensional Assessment of Interoceptive Awareness (MAIA); and the Self-as-Context (SAC) scale. A stepwise backwards linear regression and mediation analysis were performed, as well as a cluster analysis of the non-linear ANN upper hidden layer output.

**Results:** Higher levels of alexithymia were associated with increased psychological inflexibility, lower positive affect scores, and lower interoception for the subscales of “not distracting” and “attention regulation.” SAC mediated the relation between emotional regulation and total alexithymia. The ANNs accounted for more of the variance than the linear regressions, and were able to identify complex and varied patterns within the participant subgroupings.

**Conclusion:** The findings were discussed within the context of developing a SAC processed-based therapeutic model for alexithymia, where it is suggested that alexithymia is a complex and multi-faceted condition, which requires a similarly complex, and process-based approach to accurately diagnose and treat this condition.

## Introduction

Alexithymia is considered a multifaceted personality construct thought to comprise of both cognitive and affective components, and is characterized by an inability to identify and describe conscious emotions of oneself and others, as well as to regulate one's own emotions, and empathy toward others (Nemiah, 1976; Krystal, 1979, 1982; Larsen et al., 2003; Bird and Cook, 2013; Valdespino et al., 2017). Alexithymia can also be a transient state, whereby it can vary in severity given different levels of stress, anxiety, and depression (Honkalampi et al., 2000; Montoro et al., 2016), which indicates that it is related to any ongoing psychopathology.

This inability to label emotions such as sadness, fear, anger etc., may detrimentally affect individuals with alexithymia in forming effective, connected, prosocial interactions with others (Grynberg et al., 2010; Berthoz et al., 2011). This ineffective social interaction, and prosocial connectedness, may be one reason why those with alexithymia typically experience low levels of positive emotion and well-being, including happiness and life satisfaction (Timoney and Holder, 2013; Zhang et al., 2020), as well as experiencing overall negative affect (Mattila et al., 2007).

Indeed, forming positive prosocial connections has been found to be important for well-being, and lead to greater longevity. For example, in one meta-analysis, which explored 70 studies with a total of 48,673 participants, it was found that social isolation, loneliness, and living alone contributed to 29, 26, and 32% of increased risk for mortality over a 7-year period (Holt-Lunstad et al., 2015). Such evidence has prompted public health authorities to promote social connection as a priority in the US, in order to decrease risk disease morbidities and mortality (Holt-Lunstad et al., 2017).

In terms of epidemiology, there is little evidence for accurate numbers of individuals with alexithymia, with only an approximation of 10% of any given population within Western countries estimated (Berthoz et al., 2011). For example, in Finland, 9.9% of the population have ongoing alexithymia (11.9% male and 8.1% female) (Mattila, 2009). In the Eastern populations, the prevalence of alexithymia seems to be higher than in the West. In Hong Kong, for example, the number of those with ongoing alexithymia is ~37% of the adolescent population (34% male and 40% female) (Ng and Chan, 2020). However, more systematic, and recent studies are needed to differentiate between different age groups across these different regions globally. The problem of accurate figures, maybe due to the condition being multi-facet and overlapping with other conditions, such as being found to be present in a number of other problematic conditions such as substance abuse, post-traumatic stress disorder, anorexia-nervosa, and autistic spectrum disorders (ASD) (Evren et al., 2010; Beadle et al., 2013; Kinnaird et al., 2019; Passardi et al., 2019) which makes accurate diagnosis and identification difficult. So, there are clearly some gaps within the literature as to what exactly alexithymia is, what is its etiology, and whether it is a co-morbid outcome of some other condition, or the potential cause of other conditions.

In order to explore why alexithymia appears in other conditions, and to identify its underlying etiology, studies have sought to link some of the interpersonal characteristics of alexithymia, such as emotional problems, lack of empathy, and deficits of theory of mind (ToM), with that of, for example, the impaired social interactions, and similar cognitive deficits, observed in individuals with ASD. For example, it has been recognized that for both those with alexithymia and ASD, they have difficulty in recognizing emotional face expressions in others (Lord et al., 2000; McIntosh et al., 2006). Because of these similarities, some have suggested that both of these conditions relate to the same underlying emotional impairments, whereas others have suggested that emotional impairments are due to alexithymia and not autism when these conditions have been found to co-occur (Bird and Cook, 2013; Poquérusse et al., 2018).

Another area of overlap between autistic individuals and those suffering from alexithymia, relates to their specific response abilities. In one theory, Baron-Cohen extended ToM (which refers to the ability to identify the mental states of others) (Baron-Cohen, 1997), to include a response component in his empathizing-systemizing (E-S) theory (Baron-Cohen, 2009, 2016) of autism. Specifically, he suggested that there are two components to empathy which need to be explained in order to understand the social and non-social factors of the condition. The first, he suggests, ToM captures well, which is the ability to identify someone else's mental or emotional states. The second, ToM does not capture well, which is the response element to empathy i.e., the ability to respond appropriately to another person's thoughts and feelings. So, this distinction (the identification of mental states of others, and the response to mental states of others) may be an important distinguishing dimension in the study and determination of the specific etiology of autism as well as related conditions such as alexithymia.

In addition to these similarities across different conditions, there has been some interesting work in the area of psychobiology, where there has been some evidence suggesting that alexithymia is the result of a maladaptive psychophysiological system called the interoceptive pathway (Herbert et al., 2011; Brewer et al., 2016). Interoception refers to the internal representation of all bodily sensations in any given moment, and is derived from the afferent component of the autonomic nervous system (ANS) (Craig, 2002), as well as the brain's evaluation of these bodily signals (Cameron, 2001).

The interoceptive system consists of unmyelinated C and myelinated Aδ fibers, which converge signals into the spinal laminae I and II neurons and the travel through the posterior gray column of the spinal cord to the hypothalamus, anterior insular, and cingulate cortices (Pollatos et al., 2007; Craig, 2008). It receives physiological input information from viscera, thermoregulatory, nociceptive, and endocrine systems (Pollatos et al., 2007). It then integrates the inputs of varying motivational immediacy such as warmth-coldness, prickly-burning pain, taste, need to urinate, hunger, and sensual touch (Strigo and Craig, 2016). At this integration phase, the signals are then organized into primary emotional and motivational centers of the limbic system, the anterior insula, and cingulate cortices of the homeostatic sensorimotor cortex. This integrative system is activated during all emotion and motivational behavior (Murphy et al., 2003; Craig, 2014), so have an important role to play in terms of behavioral and emotional regulation (Pinna and Edwards, 2020). At this stage, it has been proposed that these centers then develop and define a meta-representation of the “self” which allow for finely-tuned regulatory responses to be formulated (Damasio and Carvalho, 2013).

The underlying neurological processes for alexithymia, specifically, however, are not well-understood, but are thought to relate to the interoceptive system. Alexithymia it is thought to be a product of a developmental dysfunction relating to reduced neural connectivity between the limbic structures, the anterior insula (AI) and anterior cingulate cortex (ACC) (Lane et al., 1997; Singer et al., 2009). Connected to these cortical pathways is the interoceptive pathway which processes awareness of bodily features through signaling via afferent neurons to brain (Craig, 2002). The interoceptive system creates homeostatic maps of the body and orchestrating regulatory responses, at the conscious level (emotional, motivations, and behavioral outcomes) as well as the unconscious level (the autonomic system) (Craig, 2014; Owens et al., 2018). So, alexithymia could be the result of problems with this underlying homeostatic mapping, regulatory responses, and meta-representation of the “self” for which Damasio and Carvalho have suggested allows for finely-tuned regulatory bodily and behavioral responses to be determined (Damasio and Carvalho, 2013).

In relation to ASD, there has been some disagreement about causality and whether a defective interoceptive system causes the symptoms of both ASD and alexithymia. One study, for example, explored the relation between oxytocin and interoception within the context of ASD, and suggested that ASD results in the failure of the interoceptive system (Quattrocki and Friston, 2014). Others have disagreed with this suggestion, and instead, have suggested that alexithymia, instead of ASD, is the result of a general failure of this interoceptive system (Brewer et al., 2015, 2016).

These types of disagreements highlight the need for more empirical work to be conducted in this area, particularly as interoception is recorded in several different ways, which capture different aspects of the outputted interoceptive system. Interoception is commonly recorded through heartbeat detection and heartbeat counting tasks via an electrocardiogram (ECG) in the form of interoceptive awareness (IA) (Thayer and Lane, 2000; Craig, 2008). However, it has also been developed within the context of a subjective questionnaire called the multidimensional assessment of interoceptive awareness (MAIA-2) (Mehling et al., 2018). This subjective version of interoceptive assessment relates to the conscious level of interoceptive experiences. As this differs to the unconscious form of heart heartbeat detection through ECG, it does not always correlate (Ring and Brener, 1996; Ceunen et al., 2013; Calì et al., 2015; Garfinkel et al., 2015; Ferentzi et al., 2018), but maybe useful in the study of alexithymia, specifically when dealing with understanding the conscious interoceptive aspects of the condition.

Mehling et al. (2018), suggested that there has been some confusion of the interoception terms being in the literature. They clarify that interoceptive accuracy (IA) relates to heartbeat detection tasks, and interoceptive awareness (IAw) which relate to those aspects which are available to conscious self-report (e.g., through the MAIA-2 questionnaire). They suggest that the self-reported IAw is comparable to interoceptive sensibility (ISen) but not interoceptive sensitivity (IS) as had been previously suggested (Ceunen et al., 2013; Garfinkel et al., 2015). For the purposes of simplicity, IAw will be used here to describe the conscious self-reporting of interoceptive experience such as recorded by the MAIA-2, in line with Mehling et al.'s suggestion.

Increased IAw has been shown to have both positive and negative outcomes. For example, increased focus on physical sensations IAw has been associated with anxiety, hypochondriasis, somatization, and hypervigilance (Paulus and Stein, 2006). However, it has been suggested that whether increased IAw leads to maladaptive or healthy outcomes, depends on the interoceptive style (Mehling et al., 2018). Mehling et al. suggest that the maladaptive form of conscious IAw which is associated with the maladaptive outcomes (e.g., of hypervigilance) is anxiety-driven conscious IAw. They suggest that with the emerging field of mindful bodily awareness, maybe a more adaptive form of IAw than anxiety-driven IAw, leading to more positive mental health outcomes. Some evidence confirm this suggestion, as mindful IAw in the form of a body scan technique (Ussher et al., 2014) has been shown to reduce pain related distress and the degree to which pain interferes with social relations. It has also shown to decrease stress states in post-traumatic stress disorder (PTSD) and associated depressive symptoms (Colgan et al., 2016).

Unfortunately, there has been only limited work in the area of mindfulness and IAw. Mindfulness practice comes in many forms, such as through mindfulness-based cognitive therapy (MBCT) (Segal and Teasdale, 2018), mindfulness-based stress reduction (Carmody and Baer, 2008; Kabat-Zinn and Hanh, 2009), and is most often defined as paying attention on purpose, in the present moment, and non-judgmentally (Kabat-Zinn, 2003). In the case of a third wave cognitive behavioral therapy, called acceptance and commitment therapy (ACT) (Hayes et al., 2006, 2009, 2011), present moment (being in the here and now) mindfulness is thought to enhance psychological flexibility. ACT also has five other key processes which also promote psychological flexibility, which include; (1) defusion–just seeing thoughts as thoughts and not buying into them; (2) acceptance—openness to painful experience rather than avoiding or suppressing it; (3) values orientation—identifying and orientating your life to what is meaningful to you; (4) commitment skills—the ongoing commitment to values; and (5) and self-as context—being the observer, through perspective-taking, to your own experience instead of being the product of it.

Studies have shown ACT (i.e., the promotion of psychological flexibility) to be effective for a variety of mental health conditions, as identified in a meta-analysis which found it to be efficacious for improving chronic pain, depression, psychotic symptoms, mixed anxiety, obsessive compulsive disorder (OCD), drug abuse, and stress at work (Öst, 2014). In a recent systematic review psychological flexibility has also been found to mediate a number of associations between ACT interventions and reduction of symptoms within psychological disorders such as anxiety and depression (Stockton et al., 2019), suggesting that psychological flexibility is indeed the active process of change in symptom reduction. In addition to this, psychological flexibility is considered a fundamental foundation to the development of positive well-being (Kashdan and Rottenberg, 2010), so is generally a useful construct to explore with many types of disorders.

The opposite of psychological flexibility is called psychological inflexibility (Hayes et al., 2004, 2006), and is characterized by experiential avoidance, believing in the literal meaning of thoughts, embodying a sense of self which is defined by the content of experience, and a lack of commitment to valued living. Some evidence of psychological inflexibility being related to the condition of alexithymia has been found, whereby adolescence with alexithymia have shown to have a combination of elevated experiential avoidance, as well as deficits in emotional regulation (Venta et al., 2013). In this study, experiential avoidance has been shown to mediate the relation between alexithymia and emotional regulation. This indicates that a large portion of the variance within the relation between regulating one's emotion and difficulty in describing ones emotional state, can be explained from the unwillingness to tolerate aversive experience. In other studies, experiential avoidance has been found to be associated with distress (Berrocal et al., 2009), as well as mediate the association between alexithymia, psychosomatic, and depressive symptoms (Panayiotou et al., 2015).

One particularly interesting construct within the ACT model, which maybe relevant to alexithymia, is called self-as-context (SAC) (Zettle et al., 2018). This is one of the six key processes within the ACT model, whereby the “self” is assumed not to be a fixed state, but instead it can become entirely flexible (less rigid) and context dependent (instead of content dependent) as an observer to experience (i.e., the perspective-taking sense of self), with increased psychological flexibility (McHugh and Stewart, 2012; McHugh, 2015).

The observing (perspective-taking) sense of self in SAC, is not just open to one's own experience, but allows the client to become open to the perspectives of others, looking through their eyes, and seeing other's experience (McHugh et al., 2019). This perspective-taking process is linked to ToM, and empathy (Decety, 2005; Harwood and Farrar, 2006; Fortier et al., 2018). In a recent systematic review (Stockton et al., 2019) it was revealed that typically SAC has not been explored as a mediator to identify process of change, and instead the other five constructs (acceptance, mindfulness, defusion, and values commitment) had been.

This is unfortunate, as perspective-taking sense of self, which consists of the interpersonal relational frames (I vs. You), temporal (Now and Then), and spatial (Here and There) (McHugh and Stewart, 2012; Edwards et al., 2017) maybe a very relevant process of change construct (mediator) in the context of alexithymia. These processes have been suggested to be important when forming connections with others, whereby prosocial connection is a basic human yearning and requirement for well-being (Hayes, 2019). Furthermore, it may be particularly relevant to alexithymia as perspective-taking may relate to the ability to identify and label one's own emotions (in a I—Now perspective observer relation), as well as the identification of emotions in others (in a You—Now perspective observer relation).

As alexithymia has been found to be related to some of the problems associated with an inflexible self, e.g., lack of empathy (Grynberg et al., 2010), affective ToM (Wastell and Taylor, 2002; Demers and Koven, 2015), and self-awareness (Moriguchi et al., 2006), these perspective-taking relations in the context of SAC, may be a useful construct to explore. Furthermore, as perspective-taking skills increase, it has been found to reduce egocentric type behavior and increase empathy driven behavior (Decety, 2005; Epley et al., 2006; Longmire and Harrison, 2018). Developing SAC relevant perspective-taking skills, therefore, maybe beneficial to those with alexithymia who have reduced empathy due to their inability to form perspective about others (Valdespino et al., 2017).

This promotion of SAC in ACT is different from other attempts to promote a positive form of “self,” such as through attempting to increasing self-esteem (Mecca et al., 1989; Myers et al., 2011). This difference is highlighted by the fact that ACT's focus is on enabling the client to see that they are not the content of their experience (i.e., developing an observer to experience sense of self), whereas other attempts to promote self in the form of self-esteem rely on inflating the clients connection to the content of experience (self as content), and in particular, specific examples of self-serving experience which inflate self-esteem.

Unfortunately, this form of inflated self-esteem has been shown to inadvertently promote problematic behaviors such as narcissism, bullying, and aggressive behavior, as a result of increased feelings of inflated sense of self (ego) (Baumeister et al., 2000). Furthermore, in some cases it has led to incidences of violent and aggressive behavior which often co-occurs alongside beliefs of superiority (Baumeister et al., 1996). Inflated sense of self, has not shown any strong causal effect for positive school achievement (Baumeister et al., 2003). Attempting to maintain high self-esteem has been linked to self-serving bias, where the individual attempts to attribute negative events to external sources, and positive experiences to themselves, in an attempt to protect their ego (Shepperd et al., 2008) as well as having developed a rigid attachment to positive self-concept (Hong et al., 1999; Blackwell et al., 2007). There are also negative outcomes in believing that the self is fixed, and cannot change, such as leading to higher levels of stress, as well as feelings of exclusion and victimization (Yeager and Dweck, 2012; Yeager et al., 2014).

Given that so little is really understood about the underlying etiological processes which cause and predict alexithymia, then this present study aims to explore some potential underlying causes which have not been previously explored such the association between SAC, IAw, and alexithymia. As alexithymia seems to involve deficits of the interoceptive pathway, and is associated with lowered well-being, increased depression, and anxiety, then this study will explore the specific associations between alexithymia, IAw, psychological flexibility, SAC, depression, anxiety, and stress, as well as positive and negative affect. Both direct and indirect (mediation) linear (through stepwise regression) will be explored, as well as and non-linear (through a deep artificial neural network; ANN) (Tu, 1996) cluster patterns to identify specific sub-group characteristics.

Given that alexithymia has been shown to relate to negative emotions, it is hypothesized that depression, anxiety, stress, and negative affect, will be positively associated with alexithymia severity, whilst positive affect will have a negative association. Also, given that those with alexithymia have reported to lack the ability to emotionally regulate, it is hypothesized that IAw, which is associated with maladaptive emotional regulation (e.g., increased anxiety), will be positively associated with alexithymia severity. Given that negative emotions are typically associated with psychological inflexibility, it is hypothesized that psychological inflexibility will be positively associated with alexithymia severity. It is also hypothesized that lower SAC will associate with the dimension EOT alexithymia as it is predicted that EOT relates to a perspective-taking (self-as-context) where the individual has formed a conceptualized form of self (which is indexed by SAC).

For the mediation analysis, as the condition of alexithymia is related specifically to deficits with ToM which in itself is related to SAC, then it is hypothesized that SAC will mediate the relation between emotional awareness IAw and alexithymia severity, whilst psychological fixability will not (or there will only be a partial mediation). Given that we predict that IAw plays an important role in alexithymia, and is related to emotional regulation (or at least emotion awareness in the case IAw), it is hypothesized that neither SAC nor psychological flexibility will mediate the relation between attention IAw and alexithymia severity (or only a partial mediation will be identified), as we predict that alexithymia is specifically an emotional regulation deficit and not an attentional one, based on limitations of recognizing emotional affect (emotional awareness) in one-self and others (i.e., through SAC orientated perspective-taking specifically).

No hypotheses were assumed for ANN as only effect sizes of the different cluster effect comparisons were reported and not a level of significance. For this section, only general patterns of the participant subgroups are of interest. As a general assumption, it is expected that the clusters will reveal highly complex and varied patterns amongst the different cluster groups. This is assumed to be due to the very complex and varied nature of alexithymia, e.g., as it is reported to appear in many other conditions such as substance abuse, post-traumatic stress disorder, and anorexia-nervosa (Berthoz et al., 2011). Certain clusters are expected, such as low effect sizes for SAC being grouped with lower effect sizes for positive affect and higher effects for depression, anxiety, and stress. It is assumed that emotion awareness IAw will cluster with SAC, but not necessarily psychological flexibility, which may form its own clusters with other non-emotion IAw sub-constructs.

## Methods

### Participants

Inclusion criteria for participation were listed as in the following: (1) participants needed to be at least 18 years of age; (2) have good ability to read English; (3) have normal to corrected to normal vision; (4) have internet access; and (5) and needed to report that that they were experiencing ongoing difficulty in labeling and describing their own emotions, which they believed was because of ongoing alexithymia. Participants were excluded if they did not meet these inclusion criteria requirements.

In total, 242 participants from the UK had responded and consented to take part in the online survey, however, 12 participant's data were removed from the study results due to them leaving after only completing part of the survey (typically just the first questionnaire). Two hundred thirty participants completed the survey. Of the total participant group, 37 fell below the TAS-20 cut-off score of 51 for possible alexithymia. However, as these participants had reported that they had problems labeling and describing emotions, and they believed they had alexithymia, they were retained in the study in line with the inclusion criteria.

### Materials

This survey was created through Qualtrics^1^. The survey included demographic questions about age, gender, and the following questionnaires:

*Toronto Alexithymia Scale (TAS-20)* (Bagby et al., 1994): This is a 20 item scale, which measures three constructs in addition to a total alexithymia score: (1) difficulty identifying feelings (DIF); (2) difficulty describing feelings (DDF); and (3) externally orientated thinking (EOT). The questionnaire is based on a five-point scale, whereby participants respond from the range 1 = strongly disagree to 5 = strongly agree. The three subscales can also be combined to give a total alexithymia score, where scores of ≥61 indicates the presence of alexithymia, 52–60 indicates possible alexithymia, ≤ 51 indicates the absence of alexithymia. Construct validity (Pinaquy et al., 2003; Larsen et al., 2006; Pike, 2013) has been found to be high for DIF (Cronbach's alpha = 0.83) and DDF (Cronbach's alpha = 0.80) subscales, but not as high for EOT (Cronbach's alpha = 0.55).

*The Depression Anxiety Stress Scales-21 (DAS-21, Short-Form)* (Lovibond and Lovibond, 1995): This is a short version which measures general ongoing (over the past week) psychological distress on three subscales (anxiety, depression, and stress). The measure has good construct validity (confirmatory factor analysis of 0.94). It also has good internal reliability as measured through Cronbach's alpha coefficients, which are 0.88 for depression, 0.82 for anxiety, 0.90 for stress, and 0.93 for the total scale (Henry and Crawford, 2005).

*Acceptance and Action Questionnaire (second version; AAQ-2)* (Bond et al., 2011): This is a seven item scale which measures general psychological inflexibility. This involves questions which ask the participant about how they accept and open up to difficult thoughts and feelings, as well as how they engage in valued behavior when difficult thoughts and feelings are present. Higher scores indicate higher psychological inflexibility. The measure has good construct validity with a Cronbach's alpha of 0.83 (Frewen et al., 2008).

*Self-as-context (SAC) scale* (Gird, 2013): This is an 11 item self-report scale and based on a seven point Likert scale with responses ranging from strongly agree to strongly disagree, where higher scores indicate higher self-as-context. The item statements include, for example; “I have a perspective on life that allows me to deal with life's disappointments without getting overwhelmed by them” and “Even though there have been many changes in my life, I'm aware of a part of me that has witnessed it all.” The scale has good construct validity with a Cronbach's alpha of 0.82.

*Multidimensional Assessment of Interoceptive Awareness (second version, MAIA-2)* (Mehling et al., 2018): This is a 32 item scale, with eight constructs, that being: (1) Noticing (noticing uncomfortable sensations in your body); (2) Non-distracting (not ignoring painful bodily sensations); (3) Not worrying (not worrying about bodily discomfort); (4) Attention regulation (being able to place attention to your body despite distractions); (5) Emotional awareness (noticing changes in your body when emotions change); (6) Self-regulation (finding a place of clam when you are feeling overwhelmed); (7) Body-listening (listening to your body for emotional states); and (8) Trusting (feeling at home in your body). The scales are rated from 0 to 5, whereby 0 = never and 5 = always. Higher scores indicate higher IAw for a particular subscale. Cronbach's alpha for all eight constructs ranged between 0.64 and 0.83. Only “noticing” (0.64) and “not worrying” (0.67) fell below the standard criterion of 0.7, indicating good overall construct validity.

*Positive and negative affect schedule-short form (PANAS-SF)* (Thompson, 2007): This is a self-report measure with 10 items which measures, in the last week, the extent to which the participant has felt positive affect (PA) (e.g., feeling attentive, alert, excited) and negative affect (NA) (e.g., feeling hostile, irritable, guilty), on a five-point scale, whereby 1 = not at all, and 5 = very much. There is good internal consistency of this scale with Cronbach's alpha showing 0.89 for the PA scale, and 0.85 for the NA scale (Crawford and Henry, 2004).

### Procedure

An advertisement was first placed on a UK alexithymia support group on Facebook outlining the study. On responding to the advertisement via email, participants were emailed a link to Qualtrics which contained an information sheet explaining the study and consent form. Once the participants consented, they completed the six questionnaires (see Materials section) as well as demographic questions.

### Ethical Statement

Ethics were approved (ethics code: 2020-2632-3880) through Swansea University's Department of Psychology Research Ethics Council (REC), which included obtaining written informed consent from all participants, right to withdraw, and a full debriefing at the end of the study in accordance with the Declaration of Helsinki.

### Data Analysis

A rule was set in Qualtrics which required participants to complete all questions, by preventing participants from progressing through the questionnaires until each section was complete, as in previous studies (Edwards, 2019). As a result, 100% of all questionnaires were completed. Descriptive and inferential statistics were all conducted within IBM's SPSS, version 26.0. For inferential statistics, four stepwise backward regressions were employed (for each of the DVs; alexithymia total, alexithymia DDF, alexithymia DIF, and alexithymia EOT) whilst for enter regressions were utilized for testing the accuracy of the ANNs estimated outputs (for alexithymia DDF, alexithymia DIF, alexithymia EOT, and the three of these combined to represent alexithymia total) against the actual alexithymia scores. Cluster analysis in the form of Ward and K-means tests were conducted for the grouping of participant predictor characteristics (see section on ANN Modeling). The ANNs themselves were conducted via Emergent software^2^, version 8.5.2 (Aisa et al., 2008).

**Figure 1:**
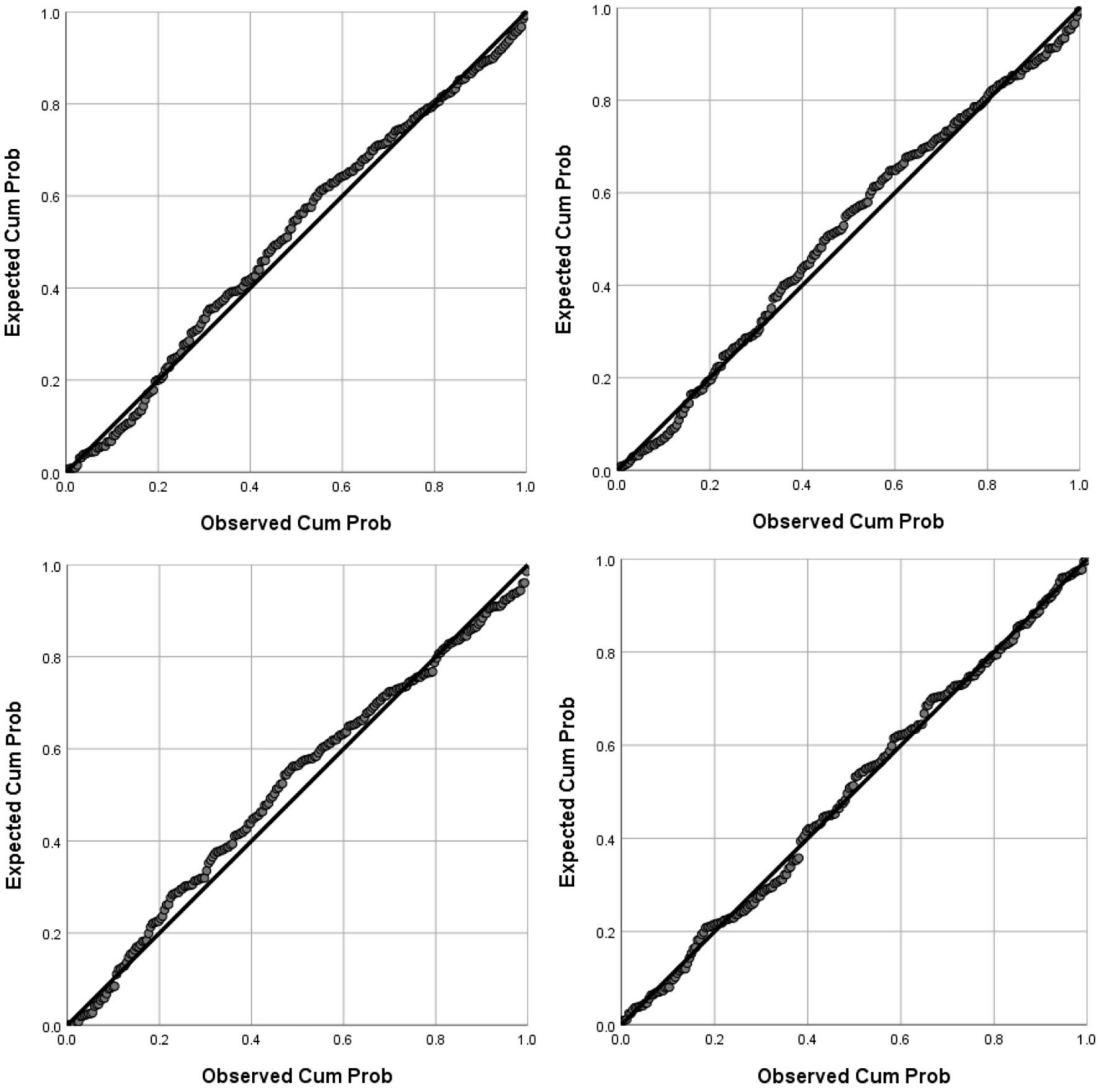
Tests of normality using the P-P plot of regression standardized residuals: Total (top left), DDF (top right), DIF (bottom left), EOT (bottom right).

**Figure 2:**
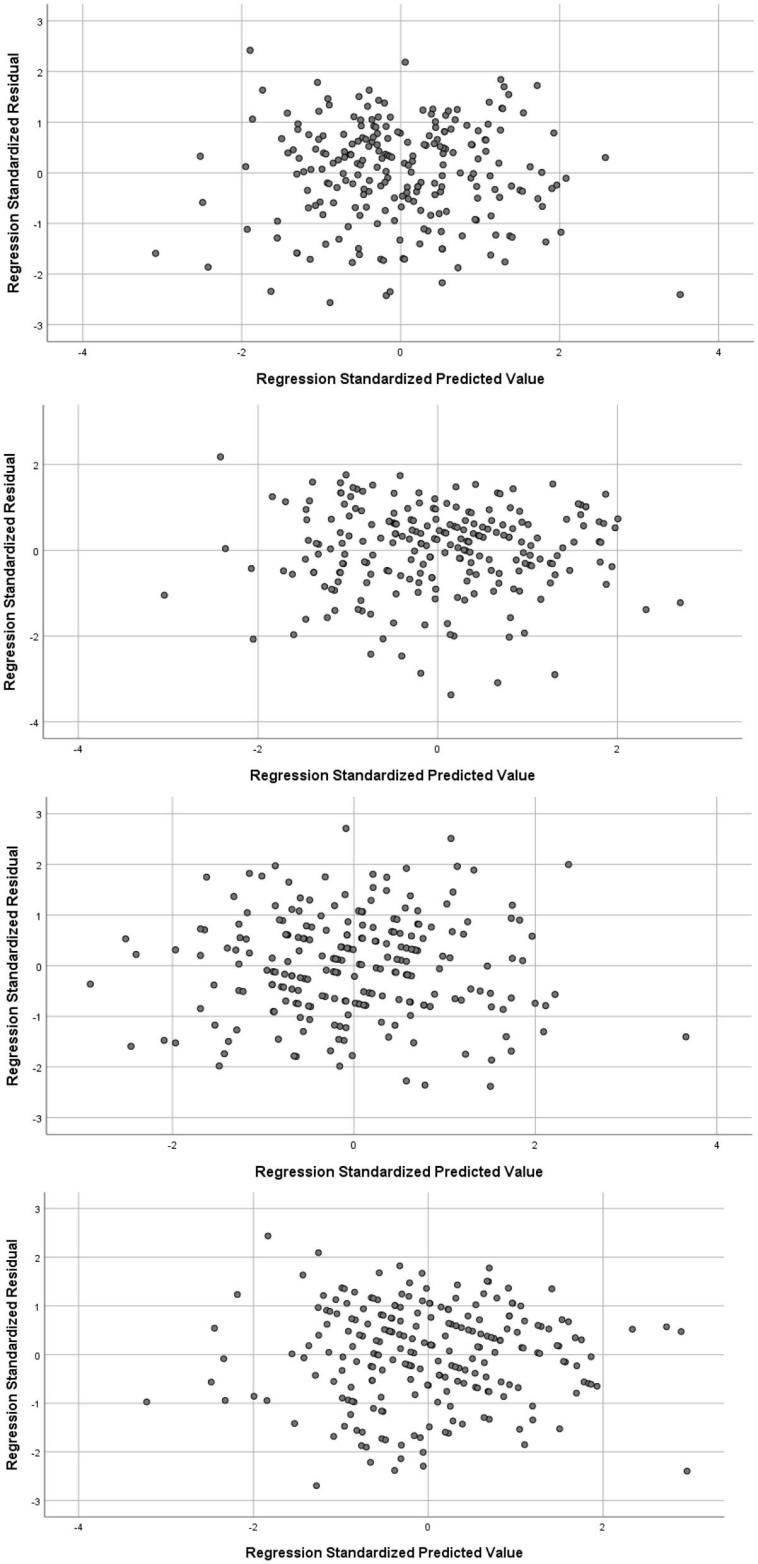
Tests of homoscedasticity with scatterplots of regression standardized residuals where the DVs are: Total (top), DDF (seconds from top), DIF (second from bottom), EOT (bottom).

Preliminary analysis were first conducted to ensure that there were no violations to the assumptions of normality, linearity, homoscedasticity, and multicollinearity. As can be seen in Figure 1, the residuals were linear and normally distributed, whilst Figure 2 shows a high degree of homoscedasticity as the residuals were spread equally. All of the correlations between the predictors were below 0.8^3^ (see **Table 3**), and the VIF scores of the coefficients were all below 10 indicating that these predictors were free from multicollinearity.

A stepwise regression was chosen over a hierarchical regression simply because not enough is known about alexithymia and related predictors in order to make assumptions about a predefined hierarchy as required for hierarchical regression models. As the hypothesis assumptions are much more tentative, instead, four separate stepwise backward regressions were employed on the 17 predictors; age, gender, three sub measures of the DAS-21 (depression, anxiety, stress); two measures of PANAS-SF (positive and negative affect); AAQII (psychological inflexibility); SAC; and the eight sub measures of MAIA-2 (IAw), against the DVs; alexithymia total, alexithymia DDF, alexithymia DIF, and alexithymia EOT. Here, with a stepwise backwards regression, the computer decides which variables are retained at each step based on a selection criteria. This selection criteria means that variables are only retained in each step if they improve the overall goodness of model fit as indexed by *R*^2^, while at the same time minimizing the complexity of the model as indexed by the Akaike information criterion (AIC), which is based on information theory (Akaike, 1974).

In order to determine whether there was sufficient power to input all 17 predictors into the stepwise backwards regression, a power analysis was conducted utilizing G^*^Power^4^ version 3.1.9.7 (Faul et al., 2007). For 17 predictors, with power set to 0.8 an a priori assumption of 146 participants was calculated as required when assuming a medium effect size based on the criteria specified by Cohen, that being 0.15 *f* ^2^ (Cohen, 1988). A larger sample was collected of 230 participants (who completed the survey), whereby a *post-hoc* analysis gave a power of 0.97 assuming a medium effect size. Given this sample, there is only a 3% chance of making a type 2 error, where the hypothesis is rejected when it should not have been (Cohen, 1988). The ANN did not require any G^*^Power analysis, as this is not a statistical test where a *p*-value is derived in the same way as regression analysis. However, as a general rule of thumb, larger samples are preferred with ANNs in order to take advantage of their flexibility, such as ability to analyse non-linearity in the data (Vach et al., 1996; Liu et al., 2017).

Four mediation analyses were conducted using the SPSS PROCESS macro (model 7) by Hayes (2013), which was set at 5,000 bootstrap samples and 95% confidence intervals to identify whether SAC and AAQ-II separately mediated the relationship between attention regulation IAw and alexithymia total, as well as emotion awareness IAw and alexithymia total.

#### Artificial Neural Network (ANN) Modeling

A backpropagation ANN was created using the Emergent software (Aisa et al., 2008). ANNs can, through broadly modeling the function of neurons, be used as a statistical tool (Detienne et al., 2003; Lowe et al., 2003, 2017). It can do this by finding regularities in relationships between the input (predictor) and output (target) variables, which is analogous to fitting a line in regression, but unlike regression it does this by assigning weights to connections between nodes of the different layers, whereby stronger associations are represented by larger weights.

This approach has several advantages over typical regression modeling (Walker and Milne, 2005). Firstly, ANNs can better handle large number of predictors, outliers, and non-linear relationships between predictors and targets. ANNs can also, and most relevant for the current study, identify groups of participants based on the similarity of their predictors-target patterns (utilizing cluster analysis of the hidden layer) which indicate which predictors are more important for particular sub groupings.

**Figure 3:**
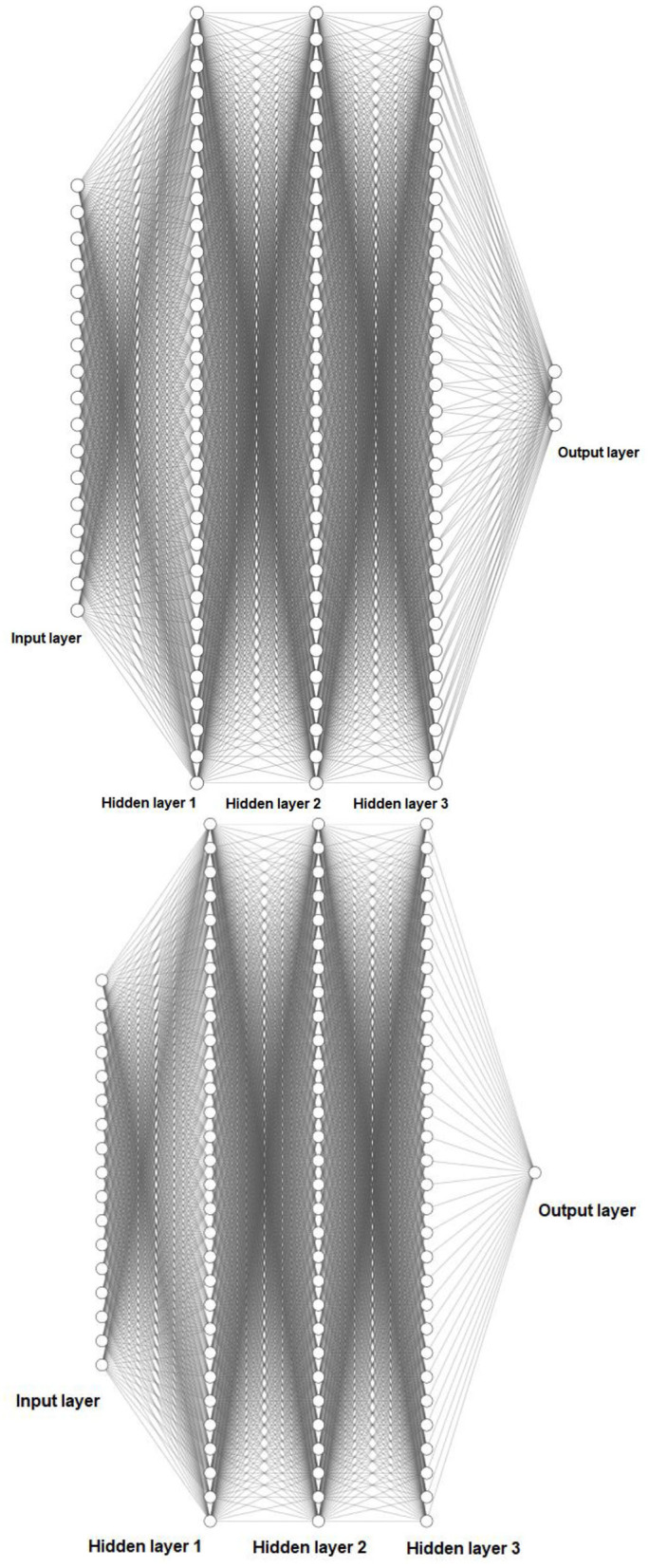
Schematic representation of (above) a deep five-layer (17 × 30 × 30 × 30 × 3) ANN as used in Network 1, and (below) a deep five-layer (17 × 30 × 30 × 30 × 1) ANN as used in Networks 2, 3, and 4.

In order to implement the ANN, first data was standardized into *z* scores in excel, before being imputed into the ANN. For Network 1, They were then organized into a series of 1 × 17 matrices for the 17 predictors. This represented the input layer with one node for each predictor and 1 × 3 for the target, i.e., three nodes to account for the three subscales of the alexithymia measure (DDF, DIF, EOT). In order to find the patterns between input and output, three hidden layers were used of 1 × 30, to ensure high predictive power (three hidden layers typically have more predictive power than a single layer), thus making the network a deep ANN (see Figure 3). Networks 2, 3, and 4 followed near identical configurations, but instead of having a 1 × 3 output layer, these included just a single (1 × 1) output layer node which represented a single output for each network (DDF, DIF, EOT) individually, as opposed to all together in a single network.

The learning rate was set to 0.001, and activity limits were set between −1 and 1, using the hyperbolic tangent (tanh) sigmoid function. For all four networks, training initialized random weights, and were constrained to between 500 and 600 epochs (which varied between networks) to prevent overfitting of the data. The learning rate was set to 0.05, while weight decay was set at 0.0001 and momentum was set as 0.1 (these were selected as they optimized the networks outcomes).

In testing for optimization, the testing split method was used, whereby the network was trained on 70% of the data and tested on the remaining 30%, which allowed for the determination of the optimal amount of training epochs, given the specific learning rates, momentum, and decay selected (Polat et al., 2008; Singh et al., 2016). As such, this method allows for the reduction of the error term, whilst preventing overfitting of the data. This means the network training is more generalizable to other data samples which use the same training data as test data.

In order to test the accuracy of the networks, the networks' estimated alexithymia outputs were linearly regressed independently using the Enter method, against the actual alexithymia outcomes, whereby if the network estimations demonstrated strong associations, i.e., through corresponding significant beta coefficients, then the network was assumed to be accurate.

#### Cluster Analysis to Identify Participant Groups and Relevant Predictors per Group

The most informative part of the network, for this current study, was the participant groupings and associated predictor characteristics, as identified through cluster analysis. This procedure was identical to that in other studies (Kimoto et al., 1990; Lowe et al., 2003, 2017) whereby cluster analysis was performed on the upper hidden layer. This layer was selected as it receives patterns from the previous hidden layers and is thus a higher level of abstraction than the previous lower hidden layers.

In order to do this, first the actual scores from the questionnaires, for each variable, were converted into *z* scores and analyzed through a two-stage cluster analysis (Milligan, 1980; Clatworthy et al., 2007). This involved a Ward (squared Euclidean distance) to identify the number of clusters (through an agglomeration schedule and dendrogram) and cluster centroids data conversion (middle of cluster), followed by a K-means method (in SPSS). The K-means require the number of clusters, and the cluster centroids (hence the Ward analysis first) to be specified. The K-means use these centroids as non-random starting seeds, which then gives the data cluster membership. This allows participant sub-groups to be identified, which reveal the relevant predictors for these subgroups.

The final step was to then compare the how each participant group differed from other groups in terms of effect sizes for relevant predictor clusters. In order to do this, each predictor variable in each group “x” was compared with the mean for that variable across all groups—e.g., mean of same predictor variable in group “y” and group “z” etc., where in this example there are three groups in total. Thus, Cohen's *d* effect sizes were calculated as $d=\;\sqrt{\frac{{\bar{x}}_{1}^{2}-{\bar{x}}_{2}^{2}\ldots etc.}{pooled\;SD}}$, whereby $Pooled\;SD=\sqrt{\frac{SD_{1}^{2}+SD_{2\ldots etc.}^{2}}{2}}$ from the means and distributions (pooled standard deviation), for each participant group and compared against the effect sizes of the other groups combined for that same variable. For gender specifically, as this was a binary variable, an odds ratio was first calculated (which is an effect size for binary variables) and then converted into a continuous effect size by dividing the odds ratio by 1.81, as suggested in other studies (Chinn, 2000).

The effect size criteria (small, medium and large effect cut of scores) were established using Cohen's (1988) criteria. Medium to large effects were considered meaningful differences whereby groupings were considered salient if comparisons differences were *d* ≥ ± 0.5 which are significant at *p* < 0.01 for *N* = 230. Small effects of *d* ± 0.35 were also reported.

## Results

### Descriptive Statistics

Table 1 shows the scoring index for the questionnaires used, whilst Table 2 shows the descriptive statistics. As can be seen from the descriptive statistics, alexithymia total mean was 61 (*SD* = 11.10), indicating the group mean was above the cut-off score for confirmed alexithymia. The mean age of the participants was 29.04 (*SD* = 9.82), and mean gender was 0.49 indicating that just under half of the participant population were male and just over half were female (as this was a binary label). This descriptive data indicates good diversity within the participant population overall. The skewness and kurtosis were all within plus or minus two except for gender (which was a simple coded binary scale) indicating a normal distribution for each variable (George and Mallery, 1999).

**Table 1:** Index of measurement score meaning.

**Outcome measure/variable**	**Meaning**
TAS-20	Higher score indicates higher alexithymia for the total and for the (three) relevant respective subsections (DDF, DIF, EOT)
DAS-21	Higher scores indicates higher depression, anxiety, stress for the (three) relevant respective subsections
AAQ-2	Higher score indicates higher inflexibility
SACS	Higher score indicates greater self-as-context
MAIA-2	Higher score indicates higher interoceptive awareness for the (eight) relevant respective subsections
PANAS-SF	Higher scores indicates greater PA and NA for the (two) relevant respective subsections

**Table 2:** Descriptive statistics and normality scores of the variables.

**Variable**	**M (SD)**	**Minimum-maximum**	**Skewness**	**Kurtosis**
Age	29.04 (9.82)	18–61	1.27	1.21
Gender	0.49 (0.50)	0–1	0.05	−2.01
TAS-20 total	61 (11.10)	26–88	−0.041	0.41
TAS-20 DDF	17.23 (4.08)	5–25	−0.48	−0.28
TAS-20 DIF	22.97 (5.47)	7–35	−0.50	0.07
TAS-20 EOT	20.80 (4.07)	11–32	0.03	−0.04
DAS-21 depression	20.12 (10.88)	0–42	0.08	−0.88
DAS-21 anxiety	14.83 (10.03)	0–42	0.44	−0.59
DAS-21 stress	20.25 (9.73)	0–40	0.06	−0.76
AAQ-2	31.67 (8.65)	10–49	−0.18	−0.65
SACS	46.01 (11.96)	11–75	−0.45	0.32
MAIA-2 noticing	2.78 (1.08)	0–5	−0.14	−0.39
MAIA-2 not distracting	2.04 (1.06)	0–5	0.14	−0.27
MAIA-2 not worrying	2.30 (1.03)	0–5	−0.03	0.02
MAIA-2 attention regulation	2.16 (1.00)	0–5	0.04	−0.28
MAIA-2 emotional awareness	2.65 (1.25)	0–5	−0.19	−0.43
MAIA-2 self–regulation	2.15 (1.14)	0–5	0.07	−0.38
MAIA-2 body listening	1.83 (1.29)	0–5	0.28	−0.59
MAIA-2 trusting body	2.20 (1.38)	0–5	0.08	−0.79
PANAS-SF PA	25.06 (7.69)	10–47	0.23	−0.31
PANAS-SF NA	27.73 (9.25)	10–48	0.06	−0.83

Table 3 shows the correlations between the variables. There was a negative correlation between age and psychological inflexibility (AAQ-II), suggesting that older participants were more psychologically flexible. There was also a positive correlation between gender and psychological inflexibility, suggesting males (who were encoded as 1) were more psychologically inflexible. Gender also positively correlated with the three subsets of alexithymia (DDF, DIF, EOT), suggesting males had a greater amount of alexithymia across these subsets. Interestingly, SAC correlated negatively with gender, suggesting that females were more able to identify themselves in the context of their experience and not content (i.e., had higher SAC).

**Table 3:** Correlations between variables.

	**1**	**2**	**3**	**4**	**5**	**6**	**7**	**8**	**9**	**10**	**11**	**12**	**13**	**14**	**15**	**16**	**17**	**18**	**19**	**20**
2	0.18^**^																			
3	−0.05	−0.06																		
4	−0.09	0.83^**^	−0.04																	
5	−0.08	0.88^**^	0.63^**^	−0.05																
6	0.06	0.72^**^	0.40^**^	0.42^**^	−0.06															
7	−0.16^*^	0.44^**^	0.34^**^	0.51^**^	0.18^**^	0.07														
8	−0.06	−0.30^**^	−0.30^**^	−0.24^**^	−0.20^**^	−0.31^**^	−0.21^**^													
9	−0.18^**^	0.32^**^	0.14^*^	0.45^**^	0.12	0.62^**^	−0.01	−0.01												
10	−0.01	−0.14^*^	−0.18^**^	−0.06	−0.13^*^	0.07	0.22^**^	0.17^**^	0.04											
11	−0.04	−0.32^**^	−0.30^**^	−0.32^**^	−0.14^*^	−0.22^**^	−0.01	−0.17^**^	0.03	0.09										
12	0.12	−0.07	−0.02	−0.12	−0.02	−0.31^**^	0.01	−0.31^**^	−0.25^**^	−0.21^**^	−0.18^**^									
13	0.03	−0.27^**^	−0.27^*^	−0.20^**^	−0.20^**^	−0.16^*^	0.31^**^	−0.06	0.45^**^	−0.03	0.08	−0.14^*^								
14	0.04	−0.14^*^	−0.17^*^	−0.01	−0.20^**^	0.10	0.31^**^	0.15^*^	0.44^**^	−0.03	−0.29^**^	0.35^**^	0.07							
15	0.09	−0.16^*^	−0.17^*^	−0.12	−0.11	−0.29^**^	0.39^**^	−0.12	0.28^**^	0.01	0.06	0.52^**^	0.41^**^	−0.02						
16	0.10	−0.19^**^	−0.22^**^	−0.11	−0.15^*^	−0.15^*^	0.39^**^	0.01	0.43^**^	0.01	−0.08	0.45^**^	0.55^**^	0.54^**^	−0.03					
17	0.10	−0.29^**^	−0.26^**^	−0.32^**^	−0.10	−0.43^**^	0.37^**^	−0.25^**^	0.18^**^	0.08	0.20^**^	0.50^**^	0.20^**^	0.49^**^	0.42^**^	−0.07				
18	0.10	−0.30^**^	−0.17^*^	−0.31^**^	−0.25^**^	−0.41^**^	0.39^**^	−0.35^**^	0.20^**^	−0.03	0.19^**^	0.46^**^	0.27^**^	0.41^**^	0.40^**^	0.48^**^	−0.09			
19	−0.17^**^	0.45^**^	0.29^**^	0.53^**^	0.22^**^	0.65^**^	−0.31^**^	0.63^**^	0.05	−0.27^**^	−0.12	−0.11	0.08	−0.13	−0.09	−0.33^**^	−0.38^**^	−0.04		
20	−0.23^**^	0.36^**^	0.15^*^	0.47^**^	0.21^**^	0.56^**^	−0.06^**^	0.69^**^	0.25^**^	−0.16^*^	−0.33^**^	−0.02	0.30^**^	0.02	0.13	−0.25^**^	−0.27^**^	0.66^**^	−0.07	
21	−0.13^*^	0.38^**^	0.22^**^	0.46^**^	0.21^**^	0.57^**^	−0.12^**^	0.71^**^	0.19^**^	−0.18^**^	−0.27^**^	−0.12	0.22^**^	−0.04	0.05	−0.27^**^	−0.37^**^	0.68^**^	0.74^**^	0.05

*1, Age; 2, Gender; 3, TAS-20 Total; 4, TAS-20 DDF; 5, TAS-20 DIF; 6, TAS-20 EOT; 7, AAQ-2; 8, PANAS-SF PA; 9, PANAS-SF NA; 10, MAIA-2 Noticing; 11, MAIA-2 Not distracting; 12, MAIA-2 Not worrying; 13, MAIA-2 Attention regulation; 14, MAIA-2 Emotional awareness; 15, MAIA-2 Self-regulation; 16, MAIA-2 Body listening; 17, MAIA-2 Trusting body; 18, SAC; 19, DAS-21 Depression; 20, DAS-21 Anxiety; 21, DAS- Stress*.

Sig, 2 tailed;

* p < 0.05;

** *p < 0.01*.

Tables 4–**7** show the outcomes form the stepwise backwards regressions. Table 4 shows the outcomes for where all 17 predictors; age, gender, three sub measures of the DAS-21 (depression, anxiety, stress); two measures of PANAS-SF (positive and negative affect); AAQII (psychological inflexibility); SAC; and the eight sub measures of MAIA-2 (IAw), were regressed against the DV (alexithymia total). For this, adjusted *R*^2^ = 0.35, and the model was significant [*F*_(7, 222)_ = 18.29, *p* < 0.001]. The same predictors were used for all of the regression model. For the second regression (see Table 5), the DV was the alexithymia subscale DDF. For this, adjusted *R*^2^ = 0.24, and the model was significant [*F*_(5, 224)_ = 15.74, *p* < 0.001]. For the third regression (see Table 6), the DV was the alexithymia subscale DIF, adjusted *R*^2^ = 0.38 and the model was significant [*F*_(5, 224)_ = 29.41, *p* < 0.001]. For the fourth regression (see Table 7), the DV was the alexithymia subscale EOT, adjusted *R*^2^ = 0.14 and the model was significant [*F*_(4, 225)_ = 9.89, *p* < 0.001].

**Table 4:** Model summary where alexithymia Total is DV.

**Variable**	**β**	**S.E**.	**Standardized β**	***t-*value**	***P-*value**
DAS-21 anxiety	0.25	0.75	0.23	3.34	=0.001
AAQ-2	0.31	0.09	0.24	3.31	=0.001
MAIA-2 not distracting	−2.53	0.58	−0.24	−4.38	<0.001
MAIA-2 attention regulation	−2.21	0.71	−0.20	−3.12	=0.002
MAIA-2 emotional awareness	−1.58	0.58	−0.18	−2.75	=0.006
MAIA-2 self-regulation	1.34	0.68	0.14	1.97	=0.05
PANAS-SF PA	−0.21	0.09	−0.14	−2.32	=0.021

**Table 5:** Model summary where alexithymia DDF is DV.

**Variable**	**β**	**S.E**.	**Standardized β**	***t-*value**	***P*-value**
Gender	−0.83	0.48	−0.10	−1.72	=0.087
AAQ-2	0.094	0.03	0.20	3.19	=0.002
MAIA-2 not distracting	−1.00	0.23	−0.26	−4.32	<0.001
MAIA-2 attention regulation	−0.80	0.25	−0.20	−3.25	=0.001
PANAS-SF PA	−0.10	0.03	−0.19	−3.04	=0.003

**Table 6:** Model summary where alexithymia DIF is DV.

**Variable**	**β**	**S.E**.	**Standardized β**	***t-*value**	***P-*value**
DAS-21 depression	0.10	0.04	0.20	2.56	=0.011
DAS-21 anxiety	0.11	0.04	0.20	2.76	=0.006
AAQ-2	0.13	0.05	0.20	2.88	=0.004
MAIA-2 not distracting	−1.01	0.28	−0.20	−3.61	<0.001
MAIA-2 attention regulation	−0.81	0.29	−0.15	−2.80	=0.006

**Table 7:** Model summary where alexithymia EOT is DV.

**Variable**	**β**	**S.E**.	**Standardized β**	***t* value**	***P-*value**
Age	0.06	0.03	0.15	2.35	=0.02
DAS-21 anxiety	0.12	0.03	0.29	3.99	<0.01
MAIA-2 emotional awareness	−85	0.23	−0.26	−3.74	<0.001
SAC	−0.039	0.02	−0.12	−1.68	=0.094

### Mediation Analysis

**Figure 4:**
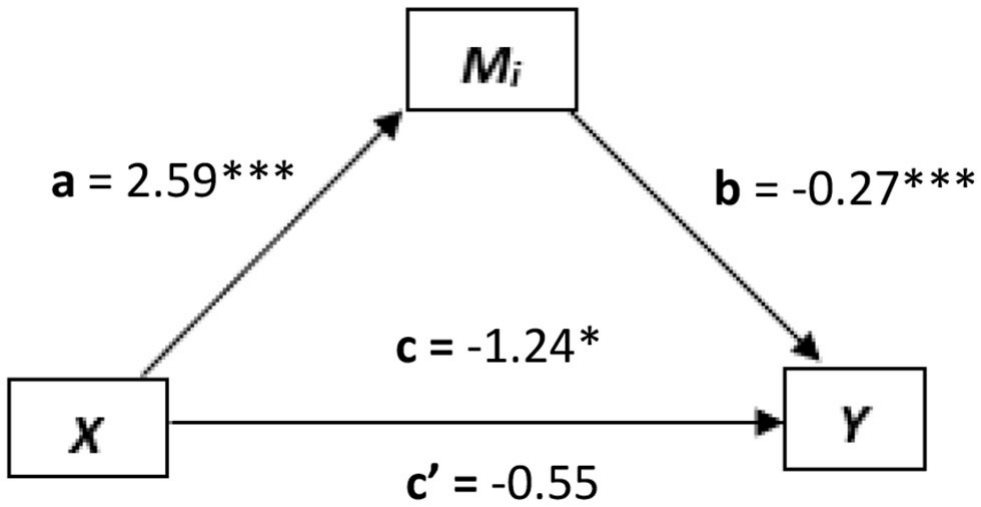
The mediated relationship, where emotional regulation interoception (*x*) predicts alexithymia total (*y*) and self as context (SAC, *M*_*i*_) mediates this relationship. *p < 0.05; **p < 0.01; ***p < 0.001.

Four separate mediation analyses were conducted. In the first, emotion awareness IAw (*x*) was explored as a predictor for alexithymia total (*y*), and SAC (*M*_*i*_) was found to mediate this relation (see Figure 4). The relationship between emotion awareness IAw and the mediator (*M*_*i*_; SAC) was significant [*a* = *t*_(228)_ = 4.27, *p* < 0.0001, *B* = 2.59], as well as the relation between *M*_*i*_ and alexithymia total [*b* = *t*_(227)_ = −4.35, *p* < 0.0001, *B* = −0.27]. The direct relation between emotion awareness IAw (*x*) and alexithymia total (*y*) before the mediator was added, was significant [*c* = *t*_(228)_ = −2.12, *p* < 0.05, *B* = −1.24]. In addition to this, the relationship between emotion awareness IAw (*x*) and alexithymia total (*y*) once the mediator was added became non-significant [*c*′ = *t*_(227)_ = −0.94, *p* = 0.35, *B* = −0.55]. As *c* to *c*′ decreased from significance to non-significance, this indicates a strong indirect mediation effect of SAC over the direct relation between emotion awareness IAw (*x*) and alexithymia total (*y*). To further support this, the confidence intervals of the indirect effect (*M*_*i*_) of *x* on *y* did not cross zero [CI = −1.34 to −0.23, *B* = −0.69] further indicating that SAC was a significant indirect mediator of the direct relation between emotion awareness IAw (*x*) and alexithymia total (*y*).

In the second mediation analysis, attention regulation IAw (x) was explored as a predictor for alexithymia total (*y*), where again, SAC was explored as the mediator (*M*_*i*_). Here, the relationships between *x* and *M*_*i*_ [*t*_(228)_ = 7.78, *p* < 0.0001, *B* = 5.46], *M*_*i*_ and *y* [*t*_(227)_ = −3.19, *p* < 0.01, *B* = −2.21], and between *x* and *y* directly [*t*_(228_) = −4.27, *p* < 0.0001, *B* = −3.01] were all significant. However, there was not a significant decrease from *c* to *c*′ (i.e., *c*′ was still significant) [*c* = *t*_(228)_ = −4.27, *p* < 0.0001, *B* = −3.01] to [*c*′= *t*_(227)_ = −2.41, *p* < 0.05, *B* = −1.87] indicating only a partial mediation. This was further evidenced, as confidence intervals of the indirect effect (*M*_*i*_) of *x* on *y* did not cross zero [CI = −1.99 to −0.32, *B* = −1.14].

**Figure 5:**
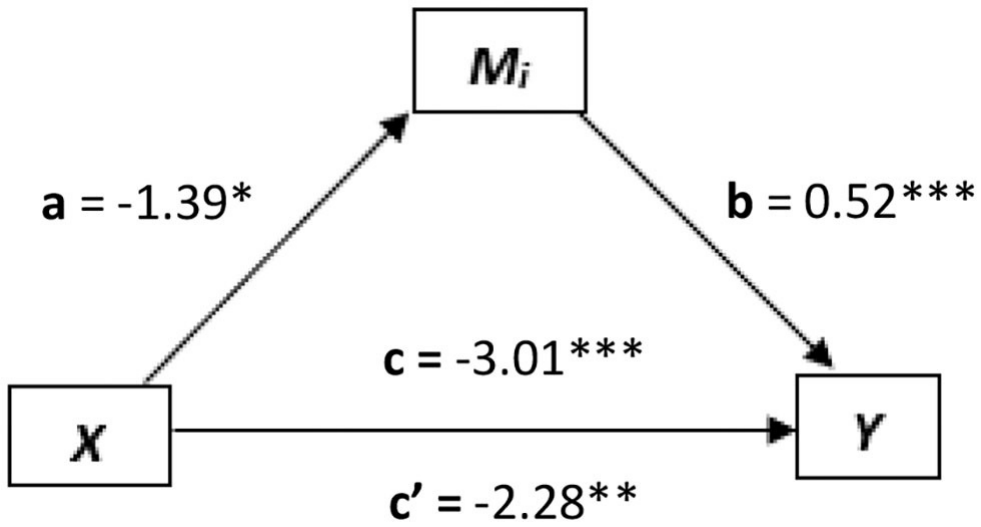
A partial mediated relationship, where attentional regulation interoception (*x*) predicts alexithymia total (*y*), and psychological flexibility (AAQ-II, *M*_*i*_) partially mediates this relationship. *p < 0.05; **p < 0.01; ***p < 0.001.

In the third mediation analysis, psychological flexibility (AAQII) was explored as a mediator (*M*_*i*_) between the predictor emotion awareness IAw (*x*) and the DV alexithymia total (*y*). However, no significant mediation between was found as the confidence intervals crossed zero [CI = −0.12 to 1.02] (see Figure 5).

In the fourth mediation analysis, psychological flexibility was explored as the mediator (*M*_*i*_) between the predictor attention regulation IAw (*x*) and alexithymia total (*y*). Here, the relationships between *x* and *M*_*i*_ [*t*_(228)_ = 7.78, *p* < 0.05, *B* = −1.39], *M*_*i*_ and *y* [*t*_(227)_ = 6.92, *p* < 0.0001, *B* = 0.52], and between *x* and *y* directly [*t*_(228)_ = −4.27, *p* < 0.0001, *B* = −3.01] were all significant. However, there was not a significant decrease from *c* to *c*′ (i.e., *c*′ was still significant) [*c* = *t*_(228)_ = −4.27, *p* < 0.0001, *B* = −3.01] to [*c*′ = *t*_(227)_ = −3.50, *p* < 0.001, *B* = −2.28] indicating only a partial mediation. This was further evidenced, as confidence intervals of the indirect effect (*M*_*i*_) of *x* on *y* did not cross zero [CI = −1.48 to −0.308, *B* = −0.73].

### Accuracy of ANN Estimates

The ANN estimated patterns of the three alexithymia subscales were entered as predictors in three regression (enter method) models, whereby the actual outcomes of these alexithymia subscales measures were entered as DVs. The following results were revealed; for DIF [Adjusted *R*^2^ = 0.77, *F*_(1,228)_ = 777.07, *p* < 0.001, β = 0.88]; for DDF [Adjusted *R*^2^ = 0.76, *F*_(1, 228)_ = 756.06, *p* < 0.001, β = 0.87]; and for EOT [Adjusted *R*^2^ = 0.69, *F*_(1, 228)_ = 516.57, *p* < 0.001, β = 0.83]. Thus, these regression models indicate that the ANN estimates were able to explain between 69 and 77% of the total variance within the DVs of the actual alexithymia subscales outcomes. Furthermore, the ANNs accounted for much more of the variance than the linear stepwise backward regressions, which only accounted for between 14 and 38% of the total variance. This perhaps indicates that the patterns between the predictors and outcomes are modeled more accurately through a non-linear approach which the ANN was able to provide.

### Clusters From Network 1

The first ANN (network 1) modeled the input (17 predictors) data against the output data of all three sub constructs together (DDF, DIF, and EOT; see Table 8). A cluster analysis on the learned patterns generated form the highest hidden layer showed that several distinct participant groupings were made (see Table 8). In the largest cluster (group 2, *N* = 115), this cluster included a large negative effect of psychological inflexibility (*d* = −0.82), suggesting that other groups were more psychologically inflexible. High not-distracting IAw positive effect (*d* = 0.91) was also found. In addition to these, gender (*d* = 0.51; i.e., more males in this group), negative affect (*d* = −0.79), depression (*d* = −0.57), and stress (*d* = −0.55), were also present as medium effects.

**Table 8:** Network 1, including DDF, DIF, EOT outcomes.

	**Effect size (*****d*****)**			
	**gp1**	**gp2**	**gp3**	**gp4**
**Predictors network 1**	**(*n =* 48)**	**(*n =* 115)**	**(*n =* 21)**	**(*n =* 46)**
Gender	0.79	0.52	0.43	—
Age	—	—	1.84	—
AAQII	1.14	−0.82	4.03	−0.36
PANAS-SF positive	—	—	1.71	0.55
PANAS-SF negative	0.87	−0.80	2.76	—
Noticing	0.61	—	1.28	—
Not distracting	—	0.91	—	—
Not worrying	−0.48	—	1.44	—
Attention	—	—	0.68	—
Emotion	0.39	—	0.78	—
Self-regulation	—	—	0.48	—
Body listening	—	—	—	—
Trusting	−0.55	—	—	0.48
SAC	−0.59	—	2.02	0.51
DAS depression	0.70	−0.57	2.42	−0.50
DAS anxiety	0.75	−0.44	0.86	—
DAS stress	1.08	−0.55	1.57	−0.40

*Displaying effect sizes (d) when comparing similar cluster groupings with the average scores across the remaining groups*.

*Sections which include a value below the minimum of small effect size (d = over ± 3.5) is depicted by a dash*.

In the second largest cluster (group 1, *N* = 48), this included high effect sizes for psychological inflexibility (*d* = 1.14), negative affect (*d* = 0.87), and high stress (*d* = 1.08). Gender featured as a medium effect (*d* = 0.79; more males in this group), noticing IAw (*d* = 0.61), and depression (*d* = 0.76), whilst having low trusting IAw (*d* = −0.55), and SAC (*d* = −0.59) which were found with negative medium effect, meaning that they were higher in the other groups combined average.

The third largest cluster (group 4, *N* = 46) included stress as a large effect (*d* = 2.10). Positive affect (*d* = 0.55), SAC (*d* = 0.51), and trusting IAw (*d* = 0.48), were found as medium effects. Psychological inflexibility (*d* = −0.36), and depression (*d* = −0.47) were found to be medium negative effects which, again, suggests that they were higher in the other groups combined average.

The smallest cluster (group 3, *N* = 21) grouped age (*d* = 1.84; i.e., more older individuals in this group), psychological inflexibility (*d* = 4.03), positive affect (*d* = 1.71), negative affect (*d* = 2.76), noticing IAw (*d* = 1.28), not worrying IAw (*d* = 1.44), SAC (*d* = 2.02), depression (*d* = 2.42), and anxiety (*d* = 0.86) together with large effects. Stress (*d* = 0.57) was found as a medium effect.

### Clusters From Network 2

The second network (Network 2) modeled the input data against the output data for just the DDF construct (see Table 9). The largest cluster (group 2, *N* = 96) only included a medium effect for gender (*d* = 0.65; i.e., more males in this group). The second largest cluster (group 3, *N* = 71), included large effects for age (*d* = 1.96; i.e., more older individuals in this group), psychological inflexibility (*d* = 2.72), positive affect (*d* = 2.33), negative affect (*d* = 2.07), noticing IAw (*d* = 1.43), not distracting IAw (*d* = 0.83), not worrying IAw (*d* = 1.32), attention regulation IAw (*d* = 1.03), emotion awareness IAw (*d* = 1.08), self-regulation IAw (*d* = 0.95), SAC (*d* = 2.64), depression (*d* = 0.85), and stress (*d* = 1.08). The third largest cluster (group 1, *N* = 46) only included gender as a medium effect, with a higher proportion of males. The smallest cluster (group 4, *N* = 17) included a large effect for gender (*d* = 0.86 i.e., more males in this group), and stress (*d* = 1.05), as well as medium effects for not worrying IAw (*d* = 0.49), and self-regulation IAw (*d* = 0.36). It also grouped a small negative effect for psychological inflexibility (*d* = −0.039).

**Table 9:** Network 2, including just the DDF outcome.

	**Effect size (*****d*****)**			
	**gp1**	**gp2**	**gp3**	**gp4**
**Predictors network 1**	**(*n =* 99)**	**(*n =* 46)**	**(*n =* 58)**	**(*n =* 27)**
Gender	0.67	0.49	0.44	0.64
Age	—	−0.43	1.97	—
AAQII	—	—	2.72	—
PANAS-SF positive	—	—	2.32	0.46
PANAS-SF negative	—	—	2.09	—
Noticing	—	—	1.36	—
Not distracting	—	—	0.77	—
Not worrying	—	—	1.39	—
Attention	—	—	0.98	—
Emotion	—	—	1.15	—
Self-regulation	—	—	0.99	—
Body listening	—	—	0.37	—
Trusting	—	—	0.58	0.37
SAC	—	—	2.58	—
DAS depression	—	—	0.95	—
DAS anxiety	—	—	0.53	—
DAS stress	—	—	1.22	1.07

*Displaying effect sizes (d) when comparing similar cluster groupings with the average scores across the remaining groups*.

*Sections which include a value below the minimum of small effect size (d = over ± 3.5) is depicted by a dash*.

### Clusters From Network 3

Network 3 modeled the input data against the output data for just the DIF construct (see Table 10). The clusters produced at the upper hidden layer were found to be nearly identical to that of Network 2. The main differences was that age did not appear in group 2 (*N* = 96), psychological inflexibility appeared in group 4 as a negative effect (*N* = 17; *d* = −0.39), and so did worrying IAw (*d* = 0.49) and self-regulation IAw (*d* = 0.36).

**Table 10:** Network 2, including just the DIF outcome.

	**Effect size (*****d*****)**			
	**gp1**	**gp2**	**gp3**	**gp4**
**Predictors network 1**	**(*n =* 46)**	**(*n =* 96)**	**(*n =* 71)**	**(*n =* 17)**
Gender	0.47	0.65	0.45	0.86
Age	—	—	1.96	—
AAQII	—	—	2.72	−0.3934
PANAS-SF positive	—	—	2.33	—
PANAS-SF negative	—	—	2.07	—
Noticing	—	—	1.43	—
Not distracting	—	—	0.83	—
Not worrying	—	—	1.32	0.49
Attention	—	—	1.03	—
Emotion	—	—	1.08	—
Self-regulation	—	—	0.95	0.36
Body listening	—	—	0.38	—
Trusting	—	—	0.65	—
SAC	—	—	2.64	—
DAS depression	—	—	0.85	—
DAS anxiety	—	—	0.50	—
DAS stress	—	—	1.08	1.05

*Displaying effect sizes (d) when comparing similar cluster groupings with the average scores across the remaining groups*.

*Sections which include a value below the minimum of small effect size (d = over ± 3.5) is depicted by a dash*.

### Clusters From Network 4

Network 4 modeled the input data against the output data for just the EOT construct (see Table 11). In the largest cluster (*N* = 99), only gender appeared as a large effect size (*d* = 0.96; i.e., more males in this group). Stress appeared as a medium effect size (*d* = 0.52), and SAC emerged as a small negative effect (*d* = −0.35) suggesting that scores were lower for this construct, in this group, when compared to the mean of the other groups combined.

**Table 11:** Network 4, including just the EOT outcome.

	**Effect size (*****d*****)**			
	**gp1**	**gp2**	**gp3**	**gp4**
**Predictors network 1**	**(*n =* 48)**	**(*n =* 115)**	**(*n =* 21)**	**(*n =* 46)**
Gender	0.96	0.83	0.59	—
Age	—	—	1.97	—
AAQII	—	−0.46	2.76	−0.42
PANAS–SF positive	—	—	2.17	0.48
PANAS-SF negative	—	−0.46	2.12	—
Noticing	—	—	1.41	—
Not distracting	—	0.52	0.62	−0.49
Not worrying	—	—	1.28	0.54
Attention regulation	—	—	1.04	0.47
Emotion	—	—	1.04	—
Self-regulation	—	—	0.77	—
Body listening	—	0.37	—	—
Trusting	—	—	0.46	0.51
SAC	−0.36	—	2.73	0.55
DAS depression	—	−0.50	1.05	−0.38
DAS anxiety	0.38	—	0.47	−0.37
DAS STRESS	0.52	−0.40	0.94	1.05

*Displaying effect sizes Effect sizes (d) when comparing similar cluster groupings with the average scores across the remaining groups*.

*Sections which include a value below the minimum of small effect size (d = over ± 3.5) is depicted by a dash*.

The second largest cluster (group 3, *N* = 58) included the large positive effect sizes; age (*d* = 1.97; i.e., more older individuals in this group), psychological inflexibility (*d* = 2.76), positive affect (*d* = 2.17), negative affect (*d* = 2.12), noticing IAw (*d* = 1.41), not worrying IAw (*d* = 1.28), attention regulation IAw (*d* = 1.04), emotional awareness IAw (*d* = 1.04), SAC (*d* = 2.73), depression (*d* = 1.05), and stress (*d* = 0.94).

For the third largest cluster (group 2, *N* = 46), only gender presented as a large and positive effect (*d* = 0.83; i.e., more males in this group), not distracting IAw presented as a medium effect (*d* = 0.52). The smallest cluster (group 4, *N* = 27), only contained stress as a large effect (*d* = 1.05), and not worrying IAw (*d* = 0.54), trusting IAw (*d* = 0.51), and SAC (*d* = 0.55), as medium effects.

## Discussion

This study sought to identify (1) whether, through a stepwise backwards regression, there were any linear relationships between the Alexithymia scale (total, and the three subscales as DVs) and the 17 predictors—age, gender, three sub measures of the DAS-21 (depression, anxiety, stress); two measures of PANAS-SF (positive and negative affect); AAQII (psychological inflexibility); SAC; and the eight sub measures of MAIA-2 (interoception); (2) whether mediation analysis could identify any indirect effects between interoception (attention regulation and emotional awareness IAw) and alexithymia total, whereby SAC and psychological inflexibility (AAQII) were explored as mediators; and (3) whether a neural network would be able to identify any non-linear patterns, whereby further cluster analysis of the upper hidden layer would reveal specific predictor characteristics within different subgroups of participants.

The findings revealed that in terms of linear associations, higher levels of total alexithymia was associated with increased psychological inflexibility, lower positive affect scores, and lower IAw for the subscales of not distracting IAw, attention regulation IAw, and emotional awareness IAw, but higher self-regulation IAw. For the DV DDF, higher DDF was associated with more females, higher psychological inflexibility, lower not distracting IAw, attention regulation IAw, and positive affect. For the DV DIF, higher DIF was associated with greater depression, anxiety, psychological inflexibility, as well as lower not distracting IAw, and attention regulation IAw. For the DV EOT, higher EOT was associated with older participants, higher anxiety, lower emotional awareness IAw, and lower SAC.

These findings supported most of the hypotheses, but not for IAw, where a negative association was largely found except in the case of self-regulation IAw. This suggest that alexithymia may relate more to a dysfunctional ability to regulate distress by connection to body sensations as opposed to simply connecting with bodily sensations. Lower SAC only associated with EOT, and this was expected given that it was predicted that SAC and EOT relate to a perspective-taking (self-as-context) form of self.

Mediation analysis showed that SAC mediated the relation between emotional awareness IAw and alexithymia total, but only partially mediated the relation between attention regulation IAw and alexithymia total. Psychological flexibility (AAQII) only partially mediated the relation between attention regulation IAw and alexithymia total, and did not mediate the relation between emotional awareness IAw and alexithymia total. This suggests further that SAC may play an important role the condition of alexithymia and in the interoceptive system, particularly in relation to emotional awareness, which is a key component to well-being (Pinna and Edwards, 2020). As such, this has implications for the types of interventions employed in treating alexithymia, whereby a process-based therapy (PBT) which target core mediators (Hofmann and Hayes, 2019) for both the diagnostic and treatment phases could be introduced. ACT maybe useful as a general-purpose intervention, but as the mediation analysis revealed, SAC was a stronger mediator for the relation between emotion awareness IAw and total alexithymia. This suggests that a bespoke intervention should target SAC processes for change (i.e., build SAC skills), particularly as the association between SAC and alexithymia was negative, indicating a low indication of SAC when alexithymia severity was high, and thus building SAC skills may reduce alexithymia severity.

In terms of the ANNs, in the first network, there were some interesting and distinct patterns. Firstly, the cluster analysis of alexithymia representing as all three subsets, revealed that there were four distinct groupings (or clusters). The largest group was characterized by high not-distracting IAw, comprising of more males, but had less psychological inflexibility, negative affect, depression, and stress, in comparison to the combined mean of the other groups. This indicates that for most individuals in this study who were largely male in this group, IAw in the form of not getting distracted was relevant, and they were relatively higher in psychological flexibility than the other groups.

Very different patterns were identified for the second largest cluster. Large effects were found for psychological inflexibility, negative affect, and high stress. This group was also largely male relative to other groups, and with depression, and noticing IAw, featuring as a medium effects. Trusting IAw, and SAC, were found as a negative medium effects. This suggests that for some individuals, they experience higher psychological flexibility problems, and have more negative emotions. This would suggest that for these individuals, traditional ACT based approach maybe effective at treating alexithymia for clients who exhibit these characteristics, as ACT specifically targets building psychological flexibility. It may also be interesting to note that noticing IAw was high, whilst trusting IAw was relatively low, meaning that the IAw subscales maybe picking up different components of alexithymia, which relate differently to those who have lower psychological flexibility and higher negative emotions.

Another group picked up more varied emotional states such as high positive effect for stress but also medium positive effects for positive affect, and trusting IAw, whilst psychological inflexibility and depression were found to be lower than in other groups combined. This suggest that for some individuals, they experienced a lot of stress in their lives but may have been experiencing relatively positive affect and were quite psychologically flexible with minimal depression. Interestingly trusting IAw appears when psychological flexibility is higher. This largely differs to the other IAw subscales, which seem present when psychological inflexibility is higher, suggesting perhaps that the IAw subscales may have different relations with psychological flexibility and within the context of alexithymia. This suggestion is also broadly supported the by the different directional IAw associations identified within the linear regressions.

The smallest cluster group were of typically older individuals than the other groups combined, with very high psychological inflexibility, but a mixture of positive affect and negative affect. These individuals were high in noticing IAw, not worrying IAw, and SAC. There were also high levels of depression, anxiety, and a medium effect for stress. It is interesting that this group has high SAC, which suggest that for a minority of individuals perspective-taking skills was not a problem, so for this group perhaps a general ACT therapeutic treatment would be more suitable than a PBT one which builds SAC skills.

For the second and third networks (these were very similar), which modeled DDF, and DIF, respectively, the most interesting aspect were found in the in the second largest group included older individuals compared to other groups, and large effects for psychological inflexibility, negative affect, noticing IAw, distracting IAw, not worrying IAw, attention IAw, emotion IAw, self-regulation IAw, as well as depression, and stress. These characterizes are very similar to the second largest cluster group for network 1 (all three alexithymia constructs combined). It is interesting to note that IAw trust did not appear, whilst the other IAw sub constructs did along with psychological inflexibility. This would suggest that general ACT maybe useful in improving these alexithymia subscales, through targeting the processes of psychological flexibility which may in turn improve IAw.

Network 4 found a unique set of patters for the EOT construct. The largest group (*N* = 99) identified only gender as a large effect, where the majority in this group were male. Stress was identified with a medium effect, whilst SAC was identified with a small negative effect. This is interesting, as SAC relates to the ability to perspective-take about oneself, and see oneself in the context of experience. This is similar in some ways to EOT which is the ability to externally orientate thinking, and is a key process in perspective-taking and therefore SAC. Therefore, the network identified an important pattern, that in those within this group, who had difficulty with external orientated thinking, had a diminished ability to form perspective-taking orientated observations about self and other. This would suggest that a PBT which focuses on building SAC ability could be useful for this particular group in reducing alexithymia severity.

The second largest group identified large positive effects for psychological inflexibility, positive affect, negative affect, IAw states of noticing, not worrying, attention regulation, emotional awareness, as well as depression, and stress. This grouping is similar to that identified by networks 2 and 3 for DDF and DIF constructs. The smallest group showed stress as a large effect not worrying IAw, trusting IAw, and SAC as medium effects. It is interesting that trusting, worrying IAw and SAC appear to together and without psychological flexibility. This suggests that SAC maybe unique with regards to alexithymia and again, this supports a case for needing to utilize a PBT approach to treating sub populations with alexithymia.

Overall, these findings are interesting, the regression models showed that there are clear positive linear relations between alexithymia severity and psychological inflexibility, depression, and anxiety, whilst negative associations with positive affect, and interoceptive states. However, the non-linear clusters show that the subgroups are very variable in identifiable characteristics of alexithymia, with very different levels of psychological flexibility, interoceptive states, positive and negative affect, SAC, depression, anxiety, and stress. This suggest that the condition of alexithymia a very complex, and multi-facet disorder, part of which may overlap with other disorders such as ASD, which share similar properties, particularly where there were limitations with SAC and EOT abilities. The interoceptive states and how these relate with SAC need further exploration in future studies.

The overall pattern of highlighted IAw states being clustered with psychological inflexibility, negative, and positive emotional states, within the ANN modeling, is consistent with the literature on interoception generally, whereby those with heightened interceptive awareness have experienced higher pain sensitivity (Pollatos et al., 2012), but also better self-regulatory capacities (Weiss et al., 2014) which may also explain why positive and negative affect components featured together with psychological inflexibility.

In terms of clinical practice, these findings are also interesting. Both traditional psychotherapy and group support for alexithymia have previously had limited success, as alexithymia patients appear to lack the skills required for psychotherapy to work effectively, such as the ability to self-reflect through accessing feelings (Ogrodniczuk et al., 2011). A review suggested that alexithymia is at least partly modifiable through therapeutic interventions, and was more successful when alexithymia symptoms were specifically targeted (Cameron et al., 2014), such as when using CBT to specifically target emotional processing (Baker et al., 2012).

The findings of this present study can build on these previous clinical findings. For example, given the finding that highly variable characteristics were identified within the cluster analyses, this suggests that more robust diagnostics are required which go beyond the standard alexithymia scales currently used today. These maybe required in order to identify the very specify needs of each individual, and which processes to specifically target therapeutically given these individual needed, such as emotional processing, ToM, or even the response processes as suggested in the E-S theory (Baron-Cohen, 2009, 2016). These could involve process-based therapy (PBT) which target core mediators (Hofmann and Hayes, 2019) and are understood more concretely through a multi-dimensional, multi-level extended evolutionary meta-model (EEMM) which provides a framework for specifying variation, selection, and retention of biopsychological properties (such as the relation between interoception and SAC) and within a therapeutic context (Hayes et al., 2020). Therefore, it is likely that bespoke, process-based interventions explored through EEMM, perhaps through a combined interoceptive, SAC, and psychological flexibility model, will need to be developed for the very specific needs of each individual suffering with alexithymia.

There are, of course, a number of limitations to this study. Being an online study, only a questionnaire-based version of the interoceptive measure was utilized. One of the problems with self-questionnaires is that they can reflect any ongoing negative affect which can lead to an increase in the intensity and frequency of their self-reported symptoms (Watson and Pennebaker, 1989). Future studies should, therefore, should seek to cross-validate these self-reported findings with an ECG measure for interoception, as well as other laboratory based neuroimaging data, such as fMIR and EEG. Such cross-methodological approaches have been argued to be extremely important for validating findings and conclusions, and in mitigating any biases in self-reporting methods (Stoyanov et al., 2012). Another potential limitation was that we included a rule in the Qualtrics, whereby participants had to complete all questions. This has the advantage of ensuring that data is one hundred percent complete, but on the other hand it could lead some participants getting frustrated, and several participants dropped out, so this approach always has benefits as well as potential limitations. Finally, this study did not take records of medication use and any co-morbidity, doing so would have allowed for greater control of these potentially confounding variables.

In summary, we have identified some interesting findings which demonstrate that alexithymia is a very complex, and multi-faceted condition, and we conclude by suggesting that clinicians and researchers should be exploring a multi-facet process-based approach at both the diagnostic and treatment phases for those who report symptoms of alexithymia. Understanding the underlying processes and interaction of interoception with SAC maybe useful to understanding how to develop bespoke treatment packages for certain sets of individuals suffering from alexithymia. Therefore, specific engagement with SAC and interoception through a PBT approach may be more beneficial than exploring an ACT approach alone. Only, then, perhaps will clinicians have a better idea for how to appropriately diagnose and treat this complex condition of alexithymia.

## Data Availability Statement

The raw data supporting the conclusions of this article will be made available by the authors, without undue reservation.

## Ethics Statement

The studies involving human participants were reviewed and approved by Swansea University Psychology Ethical Committe. The patients/participants provided their written informed consent to participate in this study.

## Author Contributions

DE designed the study, conducted the statistical analysis, and wrote the paper. RL helped with the conceptual design of the neural networks and helped with some aspects of the analysis and writing around this. All authors contributed to the article and approved the submitted version.

## Conflict of Interest

The authors declare that the research was conducted in the absence of any commercial or financial relationships that could be construed as a potential conflict of interest.

## Footnotes

^1^See here for Qualtrics: www.qualtrics.com

^2^See here for Emergent software: http://grey.colorado.edu/emergent

^3^The DVs DDF and DIF were correlated above 0.8 but this was expected as they are subscales of the same construct alexithymia total.

^4^See here for G^*^Power software: https://stats.idre.ucla.edu/other/gpower/
